# Distrontium Cerate as a Radiopaque Component of Hydraulic Endodontic Cement

**DOI:** 10.3390/ma15010284

**Published:** 2021-12-31

**Authors:** Kunlanun Dumrongvute, Sherif Adel, Takahiro Wada, Nobuyuki Kawashima, Chinalai Piyachon, Hiroshi Watanabe, Tohru Kurabayashi, Takashi Okiji, Motohiro Uo

**Affiliations:** 1 Department of Pulp Biology and Endodontics, Graduate School of Medical and Dental Sciences, Tokyo Medical and Dental University, 1-5-45 Yushima, Bunkyo-ku, Tokyo 113-8549, Japan; kunlanun_d@hotmail.com (K.D.); adelendo@tmd.ac.jp (S.A.); kawashima.n.endo@tmd.ac.jp (N.K.); okijendo@tmd.ac.jp (T.O.); 2 Department of Conservative Dentistry and Prosthetics, Faculty of Dentistry, Srinakharinwirot University, 114 Sukhumvit 23, Wattana, Bangkok 10110, Thailand; chinalai@g.swu.ac.th; 3 National Research Centre of Egypt, Department of Restorative Dentistry and Dental Materials, Oral and Dental Research Division, El Buhouth Street, Dokki, Cairo 12622, Egypt; 4 Department of Advanced Biomaterials, Graduate School of Medical and Dental Sciences, Tokyo Medical and Dental University, 1-5-45 Yushima, Bunkyo-ku, Tokyo 113-8549, Japan; wada.abm@tmd.ac.jp; 5 Department of Oral and Maxillofacial Radiology, Graduate School of Medical and Dental Sciences, Tokyo Medical and Dental University, 1-5-45 Yushima, Bunkyo-ku, Tokyo 113-8549, Japan; hiro.orad@tmd.ac.jp (H.W.); kura.orad@tmd.ac.jp (T.K.)

**Keywords:** compressive strength, distrontium cerate (2SrO·CeO_2_), flowability, hydraulic endodontic cement, radiopacity

## Abstract

This study aimed to synthesize distrontium cerate (2SrO·CeO_2_: S_2_Ce) and evaluate its properties as an alternative component of the endodontic cement. S_2_Ce cement was prepared through calcination of strontium hydroxide and cerium carbonate. Subsequently, the crystal phase was confirmed using X-ray diffraction. S_2_Ce cement exhibited a rapid setting time (121 min) and achieved a high compressive strength (72.1 MPa) at 1 d after mixing, comparable to the compressive strength of a commercial mineral trioxide aggregate (MTA) cement (ProRoot MTA) after 28 d post mixing. However, the compressive strength decreased after 28 d of storage when the W/P ratio was 0.30–0.40 (*p* < 0.05). Ion dissolution test of the S_2_Ce cement showed that strontium ions were released after immersion in water (5.27 mg/mL after 1 d), whereas cerium dissolution was not detected. S_2_Ce exhibited approximately three times higher radiopacity (9.0 mm aluminum thickness equivalent) compared to the commercial MTA (*p* < 0.05). These findings suggest that S_2_Ce is a possible component for hydraulic endodontic cement that demonstrates a rapid setting and high radiopacity.

## 1. Introduction

Mineral trioxide aggregate (MTA) has been used in pulp capping and retrograde filling and as a perforation repair material since the early nineties [1,2], owing to its remarkable capacity to induce hard tissue formation at the site of its application, such as in the case of direct pulp capping [3,4,5,6] and after full pulpotomy [7]. The main ingredient of MTA is tricalcium silicate (3CaO·SiO_2_: C_3_S), which is detectable in low contrast in X-ray radiography [8]. Therefore, heavy element oxides are added to commercial MTA cements to improve radiopacity. Bismuth oxide is a typical radiopacifier additive to MTA, which constitutes approximately 20% of the MTA composition [9]. However, the decrease in the compressive strength and tooth discoloration caused by bismuth oxide addition is of concern [10,11,12,13]. Therefore, the development of a novel cement ingredient, which has a high radiopacity and contributes to cement hydration and setting, can enhance the usability of MTA. 

Previously, the authors developed strontium aluminate (3SrO·Al_2_O_3_: S_3_A) as a self-setting and radiopaque component for the MTA cement [14]. Strontium is an alkaline earth element like calcium and is naturally present in bones and teeth. Sr ions are rapidly incorporated into tooth hydroxyapatite from the surrounding fluid, and their effects on improving the acid resistance and remineralization have been reported [15,16,17,18,19,20]. In addition, Sr is a heavier element than Ca; therefore, the enhancement of radiopacity is expected [21]. Experimentally derived S_3_A cement exhibited a radiopacity similar to that of a commercial MTA cement (ProRoot MTA; Dentsply Tursa Dental, Johnson City, TN, USA) without the addition of a radiopacifier. While S_3_A exhibited a faster setting time, it demonstrated a lower compressive strength compared to commercial MTA. As the main component of endodontic cements, S_3_A exhibited sufficient radiopacity; however, the compressive strength is not sufficient. A mixture of S_3_A and C_3_S, which was the main component of MTA, exhibited an improved compressive strength, but the radiopacity of S_3_A was reduced to an insufficient level. Therefore, the development of novel cement components that can achieve both higher radiopacity and better mechanical properties than commercial MTA is required.

The synthesis and properties of distrontium cerate (2SrO·CeO_2_: S_2_Ce) have been reported for use as a matrix for rare-earth doping phosphors [22,23]. The possibility of using this compound as a component of the dental cement has not yet been investigated. Cerium is a lanthanoid and an adjacent element of lanthanum. Lanthanum oxide is widely used as a contrast agent in dental composite resins. Therefore, a higher radiopacity than that of S_3_A is expected for S_2_Ce. Additionally, bioactive effects may be present for both Sr and Ce. For example, CeO_2_ nanoparticles have been shown to promote the osteogenic differentiation and mineralization of osteoblasts [24], as well as the X-ray irradiation protection for cells by promoting the reduction of the reactive oxygen species [25]. If S_2_Ce exhibits similar hydration and setting behavior to those of calcium silicate, the main ingredient of MTA, S_2_Ce would be a promising candidate to improve the radiopacity of MTA cements without adding bismuth oxide.

The aim of this study is to synthesize S_2_Ce as a cement component that provides sufficient radiopacity, and to investigate the properties of S_2_Ce in terms of the setting time, compressive strength, relative flowability, and radiopacity.

## 2. Materials and Methods

### 2.1. Preparation of S_2_Ce and Experimental Cements

S_2_Ce cement was prepared according to a previously reported method [14]. Briefly, strontium hydroxide octahydrate (Sr(OH)_2_·8H_2_O; Kanto Chemical Co. Inc., Tokyo, Japan) and cerium carbonate octahydrate (Ce_2_(CO_3_)_3_·8H_2_O; Kanto Chemical Co. Inc., Tokyo, Japan) were mixed at a molar ratio of 4:1, heated for 2 h at 180 °C to allow the hydration water to evaporate, and ground using an agate mortar. The obtained powder was calcined at 800, 1000, and 1200 °C for 4 h and ground again in the agate mortar. X-ray diffraction (XRD, Miniflex, Rigaku Corp., Tokyo, Japan) was used to identify the crystal phase of the obtained powder at a scanning speed of 2°/min. 

As a representative commercially available MTA product, white ProRoot MTA was used for comparison with the experimental cement.

The S_2_Ce cement was mixed with distilled water (DW) at water/powder (W/P) ratios from 0.25 to 0.5. ProRoot MTA was mixed with DW at a W/P ratio of 0.33, according to the instructions of the manufacturer.

### 2.2. Scanning Electron Microscopy (SEM) Observation

The microstructure of the S_2_Ce powder and the cement specimen cured at 37 °C with 100% relative humidity (RH) for 28 d after mixing were observed using SEM (TM4000Plus, Hitachi High-Technologies Corp., Tokyo, Japan); the observation was performed under an acceleration voltage of 15 kV and magnification of 1000.

### 2.3. Setting Time Evaluation

The setting time was determined according to the ISO 9917-1 (2007) specifications. The cement mixture was filled into a plastic mold with an inner diameter of 10 mm and a height of 2 mm placed in a sealed container and maintained at 37 °C with 100% RH. Each specimen was subjected to an indentation test using a Vicat needle (diameter: 1 mm) with a mass of 400 g. Indentations were made periodically until the needle failed to create a complete circular indentation in the cement. The setting time was calculated as the time elapsed from the beginning of cement mixing to the absence of indentation. Three replicates of each material were tested, and the results were averaged.

### 2.4. Compressive Strength Evaluation

The compressive strength was determined according to the ISO 9917-1 (2007) specifications. The cement mixture was filled into a separable cylindrical stainless-steel mold with inner dimensions of 4 mm diameter and 6 mm height. The mold was then covered on both sides with glass plates and maintained at 37 °C with 100% RH. The specimens were divided into two groups. One half was kept for 1 d, and the other half was kept for 28 d. After the given period, the specimen was carefully removed from the mold and its diameter and height were measured using a screw micrometer (QuantuMike series 293; Mitutoyo Corporation, Kawasaki, Japan). The compressive strengths were determined using a universal testing machine (EZ-LX, Shimadzu Corporation, Kyoto, Japan) at a test speed of 0.75 mm/min. Five replicates for each material were tested, and the results were averaged.

### 2.5. Relative Flowability

To estimate the rheological behavior of S_2_Ce cement, the relative flowability was evaluated according to a previously reported method [26]. A total of 0.06 g of powder for each cement was mixed with DW at the corresponding W/P ratio. The mixture was placed on a glass slab using a plastic ring of 6 mm diameter and 1 mm thickness. A precision balance (AG245, Mettler Toledo, Greifensee, Switzerland) was used to measure the mass of the cement. After removing the ring, a second glass slab was placed on top of the cement, and a weight of 1 kg was applied for 60 s at room temperature (24 °C). The surface of the spread cement was then digitized using a flat-bed scanner (ApeosPort-IV C3375, Fuji Xerox, Tokyo, Japan) at a resolution of 600 dpi, and the surface area was calculated using an image analysis software (ImageJ, Ver. 1.52K, National Institute of Health, Bethesda, MD, USA). The relative flowability was calculated as follows:

$$Relative\;Flowability=\frac{area\;\left({\mathrm{mm}}^{2}\right)}{weight\;of\;paste\;\left(g\right)}.$$

Five replicates of each material were tested, and the results were averaged.

### 2.6. Ion Dissolution from Cured Cement

The mixed cement specimen was filled into a plastic mold with an inner diameter of 10 mm and a height of 2 mm and cured in a sealed container maintained at 37 °C with 100% RH for 24 h. After curing, the cement-filled mold was immersed in 10 mL of DW in a sealed plastic container and kept for 1 and 3 d, respectively. The immersed solution was centrifuged at 3000 rpm and filtered with a syringe filter (pore diameter of 0.2 μm; Advanced Microdevices Pvt. Ltd., Ambala Cantt, India) to remove the cement particles. Then, the solutions were used for the analysis of dissolved ion concentrations. Sr and Ce contents were quantified using inductively coupled plasma atomic emission spectroscopy (ICP-AES: Spectro Arcos, Hitachi High-technologies, Tokyo, Japan). Sr (1000 ppm, Nacalai Tesque Inc., Kyoto, Japan) and Ce (1000 ppm, FUJIFILM Wako Pure Chemical Corp., Osaka, Japan) standard solutions were used as standard solutions for ICP-AES analyses.

### 2.7. Radiopacity

Radiopacity was estimated according to the ISO 6876 (2012) specifications. Disk-shaped specimens with the thickness of 0.4–0.5 mm were fabricated by filling the cement mixture into plastic molds. The thicknesses of the specimens were measured with a screw micrometer. The specimens and an aluminum step wedge (with a thickness of 1–10 mm) were placed on an X-ray imaging plate and exposed to X-rays with a dental X-ray equipment (MaxiX, Morita Corp., Kyoto, Japan) under the following conditions: voltage of 70 kV, current of 7 mA, exposure time of 0.63 s, and 15 cm distance between the imaging plate and cone end. An imaging plate reader (Carestream CS 7600, Carestream Health Japan Co., Ltd., Tokyo, Japan) was used to obtain the radiographs. The mean grey value (MGV) of the specimen was determined using image analysis software (ImageJ, Ver. 1.52K, National Institute of Health, Bethesda, MD, USA), and the aluminum thickness equivalent was calculated by normalizing the specimen thickness. Three replicates of each material were tested.

### 2.8. Statistical Analysis

One-way analysis of variance (ANOVA), followed by Tukey’s post-hoc test, was used to analyze the compressive strengths of samples with different W/P ratios after 1 d or 28 d from cement mixing. The compressive strengths of 1 d and 28 d samples at different W/P ratios were analyzed by an F-test followed by a *t*-test. The radiopacity of S_2_Ce and MTA was also analyzed by an F-test followed by a *t*-test. One-way ANOVA followed by the Dunnett post-hoc test was used to analyze the flowability of samples with different W/P ratios, and samples with a W/P ratio of 0.3, which showed the lowest flowability among the analyzed samples, were used as controls. The statistical significance was set at *p* < 0.05. 

## 3. Results

### 3.1. XRD Analysis and Setting Time of the Synthesized S_2_Ce Powder

**Figure 1:**
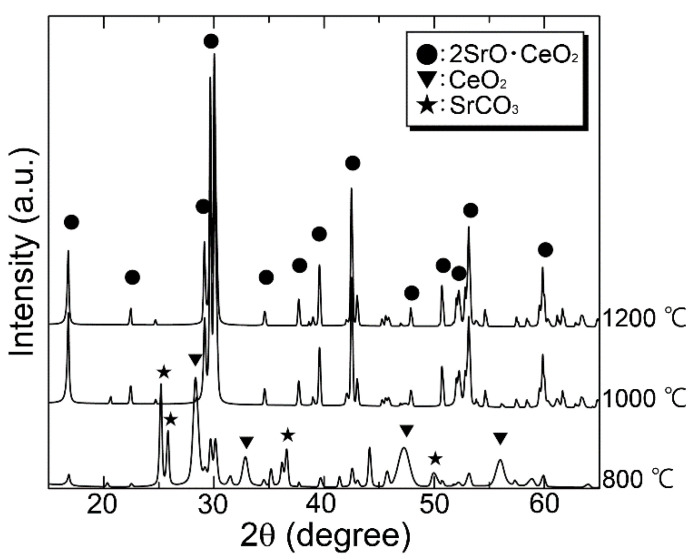
XRD pattern of S_2_Ce powders calcined at various temperatures.

The XRD pattern of the synthesized S_2_Ce powder is shown in Figure 1. All observed peaks could be assigned to those of the S_2_Ce for the powders calcined at temperatures higher than 1000 °C. The powder calcined at 800 °C exhibited peaks assigned to SrCO_3_ and CeO_2_, indicating that the reaction of these two components did not occur. Thus, calcination temperatures higher than 1000 °C are necessary for S_2_Ce synthesis. Therefore, the powder calcined at 1200 °C was used in the following studies. The setting time of S_2_Ce was 121 ± 8 min at a W/P ratio of 0.4.

### 3.2. SEM Observation

**Figure 2:**
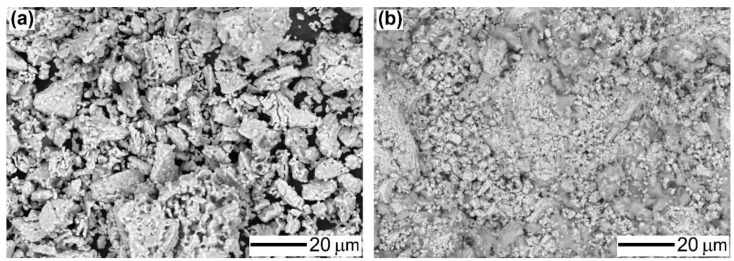
SEM images of S_2_Ce powder (**a**) and S_2_Ce cement (W/P = 0.4) at 28 d after mixing (**b**).

Figure 2 shows the SEM images of S_2_Ce powders (a) and the cement after 28 d curing with W/P = 0.4 (b). The S_2_Ce powders consisted of small primary particles of a few μm in diameter and secondary particles, which had irregular shapes with sizes up to 20 μm. The S_2_Ce cement showed that small precipitates were filled among the resource particles.

### 3.3. Compressive Strength

**Figure 3:**
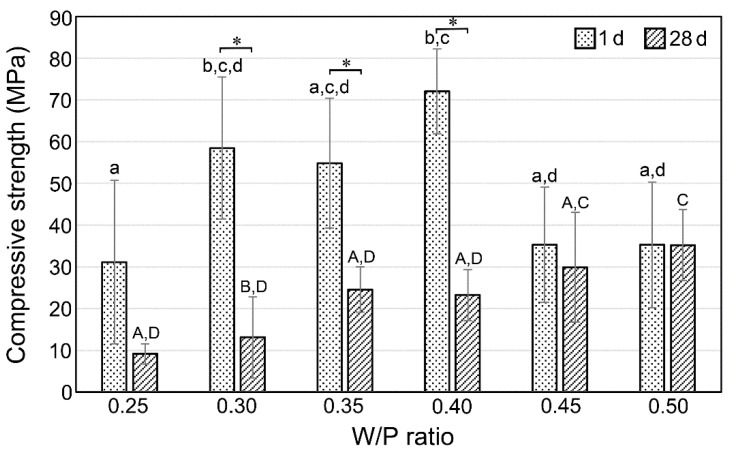
Compressive strengths of S_2_Ce cements after 1 d and 28 d from cement mixing. Columns with different letter(s) (lower case (a–d) for 1 d group and capitals (A–D) for 28 d group) are significantly different (*p* < 0.05). Asterisk (*) represents significant differences between the 1 d and 28 d maturing periods of the samples with the same W/P ratio.

The compressive strength evaluated for the S_2_Ce cement prepared with various W/P ratios at 1 d and 28 d after mixing is shown in Figure 3. After 1 d curing, specimens mixed with the 0.25 W/P ratio exhibited a significantly lower compressive strength than those mixed with the 0.30 W/P ratio. The mixture prepared at 0.25 W/P ratio had low flowability. Therefore, the preparation of test specimens by filling the mixture densely into the metal mold was challenging. Thus, the prepared specimens might have contained defects that resulted in the low compressive strength. The compressive strength was not significantly different in specimens mixed with the W/P ratio of 0.30–0.40. However, specimens mixed with the W/P ratio of 0.45–0.50 exhibited lower values than those mixed with the W/P ratio of 0.40. Specimens mixed after 28 d with the W/P ratio of 0.30–0.40 exhibited lower compressive strengths compared to those mixed after 1 d with the same W/P ratio.

### 3.4. Relative Flowability

**Figure 4:**
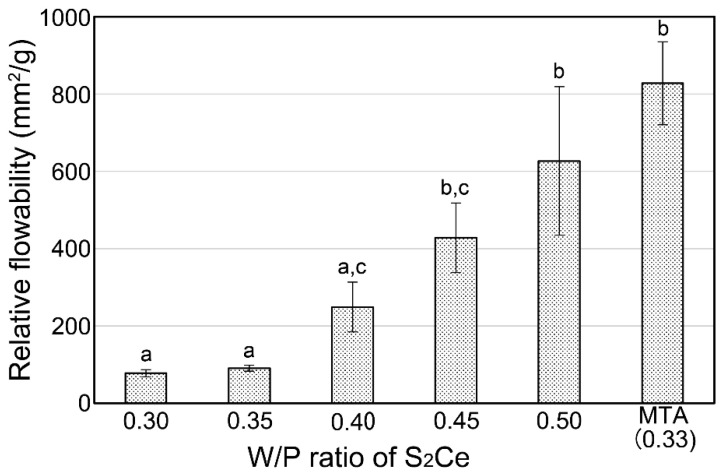
Relative flowability of the S_2_Ce cement paste mixed with various W/P ratios. Result of MTA was derived from a previous report [14]. Columns with different letter(s) (a–c) are statistically different (*p* < 0.05).

Figure 4 shows the relative flowability of S_2_Ce cements mixed with various W/P ratios. The cement paste mixed with the 0.25 W/P ratio did not spread, and its flowability was not measurable. The flowability was improved by increasing the W/P ratio, and values similar to those of MTA presented in our previous report [14] were obtained at ratios ≥ 0.45.

### 3.5. Ion Dissolution from the Cured Cement

As shown in Table 1, the Sr concentration in the surrounding water, leached from the S_2_Ce cement mixed with the W/P ratio = 0.4, was 5.27 mg/mL (195 mM) after 1 d of immersion, and the concentration did not significantly increase after 3 d of immersion. In contrast, Ce was not detected at both 1 d and 3 d of immersion. Therefore, Sr rapidly and selectively dissolved into the surrounding water in the early stage and the water would be almost saturated with Sr ion. 

### 3.6. Radiopacity

**Figure 5:**
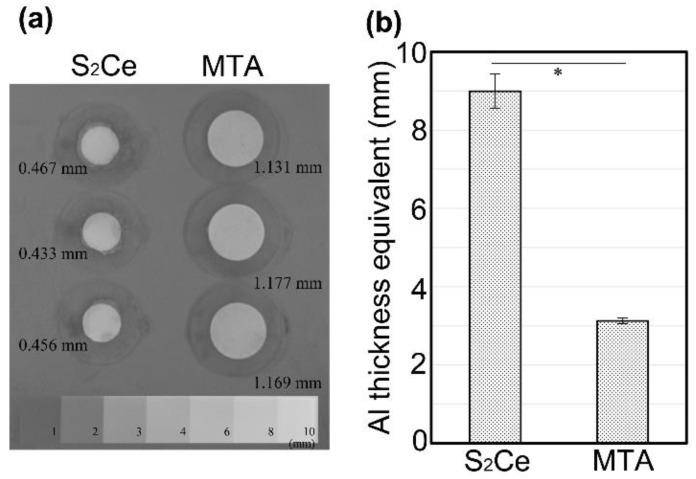
(**a**) Digital radiographs of S_2_Ce and MTA cement specimens and an Al step-wedge. Numbers in the photo show the thickness of the specimens. (**b**) Radiopacity values of cements are presented as Al thickness equivalent (mm). Asterisk (*) represents significant difference between S_2_Ce and MTA (*p* < 0.05).

The S_2_Ce cement sample (W/P = 0.4) with a thickness of 0.4–0.5 mm exhibited a similar radiographic contrast, compared to the commercial MTA cement (ProRoot MTA) with a thickness of 1.1 mm, as shown in Figure 5a. The normalized Al thickness equivalence of the S_2_Ce cement was 9.0 ± 0.4 mm, which was roughly three times higher than that of the commercial MTA cement.

## 4. Discussion

Tricalcium silicate (C_3_S), the main ingredient of MTA, exhibits X-ray absorption properties similar to those of the tooth [8]. Therefore, heavy element oxides (e.g., bismuth oxide) are added as radiopacifiers. However, some negative effects of bismuth oxide, including tooth discoloration and setting deterioration, have been reported [10,11,12,13]. Therefore, we investigated an alternative cement component consisting of a heavier element complex oxide. In a previous study [14], we reported that S_3_A exhibited a radiopacity similar to that of commercial MTA (ProRoot MTA), while the compressive strength of S_3_A was not satisfactory for use as the main ingredient of the cement. In this study, we developed a novel component, S_2_Ce, which consisted of heavier element oxides than S_3_A, and its properties were investigated.

S_2_Ce was successfully synthesized from strontium hydroxide and cerium carbonate by calcination at 1200 °C. Compared to the calcination temperature of Portland cement, which is typically 1400–1500 °C, S_2_Ce could be synthesized at lower temperatures. The experimental S_2_Ce powders had larger particle sizes and irregular shapes, similar to the previously developed S_3_A powders [14]. 

In a previous study, we reported a setting time of 90.7 min for S_3_A and 153 min for C_3_S, the main ingredient of MTA [14]. In this study, the setting time of S_2_Ce was shorter (121 ± 8 min). Thus, Sr-containing complex oxides would exhibit a shorter setting time compared to calcium silicate-based cement ingredients. A long setting time of 261 min [27] has been reported for commercial MTA (ProRoot MTA) and is considered a major drawback of this material. The shorter setting time of S_2_Ce might help to shorten the setting time of MTA.

The compressive strength of S_2_Ce mixed with W/P = 0.4 achieved 72.1 MPa after 1 d of maturation, as shown in Figure 3. The compressive strength of commercial MTA (ProRoot MTA) is reported as 27.41 MPa after 1 d [28], and it increases to 86.02 MPa after 28 d [29]. Therefore, S_2_Ce exhibited fast setting, and a higher compressive strength was achieved after 1 d post mixing. However, S_2_Ce exhibited a significant reduction in the compressive strength at 28 d after mixing in samples with W/P ratios between 0.3 and 0.4, which could be associated with certain degradation during the curing period, such as the dissolution behavior. Thus, a fast setting is expected for S_2_Ce, while an improvement in stability is desirable for this material.

The relative flowability of the S_2_Ce mixture was similar to that of commercial MTA (ProRoot MTA) with W/P ratios ≥ 0.45. However, the compressive strength was low in those W/P ratios, and the flowability with W/P = 0.4, which is the optimum condition to obtain best compressive strength, was quite lower than that of commercial MTA. In this study, S_2_Ce powders were prepared through manual grinding by using an agate mortar. Therefore, coarse granules with irregular shapes resulted in low flowability.

The dissolution behavior could be considered as one of the reasons that the compressive strength of S_2_Ce decreased after 28 d of maturation. The solubility of strontium oxide in water is reported to be 6.9 mg/mL for cold water (30 °C), while cerium oxide is reported to be insoluble in water [30]. As shown in Table 1, the Sr content in the immersed water was 5.27 mg/mL after 1 d immersion that was close to the above solubility limit. Therefore, Sr dissolved rapidly in the surrounding water until saturation. In contrast, Ce has no solubility in water; therefore, only Sr dissolved selectively. This rapid dissolution of Sr considerably decreased the compressive strength after 28 d, which was maintained at 37 °C and 100% RH. The specimens were not in direct contact with the liquid water, while the condensed water from the vapor on the specimen surface might wash out Sr selectively; thus, the cement would possibly be weakened. 

The effect of extraction solution from S_3_A on mouse dental papilla cells was studied to elucidate the effect of Sr released from the cement. The extraction solution containing Sr and Al ions exhibited higher mineralized nodule formation and mRNA expression of bone morphogenic protein 2, osteocalcin, and osteopontin than those of the MTA extract [31]. Therefore, a similar pro-mineralization effect could be expected for S_2_Ce cement, which we will investigate in future studies.

The flowability of the S_2_Ce cement (Figure 4) was lower than those of S_3_A and commercial MTA in a previous report [14]. Even with the largest W/P ratio (0.5), the flowability of the S_2_Ce cement was lower than that of the commercial MTA (W/P = 0.33) achieved in the previous report. The large particle size and irregular shape of S_2_Ce are the main reasons for the low flowability. Flowability is one of the important factors that dominate the handling properties of cement. Therefore, further studies on the particle size and shape control are required.

Regarding the radiopacity, as shown in Figure 5, the S_2_Ce cement exhibited three times higher contrast on X-ray radiography compared to the commercial MTA (ProRoot MTA), which contained bismuth oxide as the contrast agent. The typical dental X-ray emission spectrum, operated at 80 kV, has a spectrum peak at 34 keV, and the full width half maximum is approximately at 23 to 58 keV [32]. Therefore, the irradiated X-ray contains a higher energy X-ray component than that at 40 keV. The X-ray absorption by the atoms exhibits the discontinuous increase at certain energies called “absorption edges;” therefore, most efficient absorption occurs just above the absorption edges. The absorption edge energy is an atom characteristic. The absorption edge energies of the K-shell electrons of Sr and Ce are 16.1 and 40.4 keV, respectively [30]. Therefore, Sr exhibits high absorption for X-rays with energies of 16 keV or more; however, high-energy X-rays at energies higher than 40 keV are easily transmitted. In contrast, the K-edge of Ce is close to the spectrum peak of typical dental X-ray emission; therefore, Ce efficiently absorbs the higher energy component of the irradiated X-ray. This is the reason for the high radiopacity of S_2_Ce.

In this study, we synthesized S_2_Ce and demonstrated the feasibility of using it as a component of MTA. S_2_Ce exhibited a fast setting time as well as a high initial compressive strength. The radiopacity of S_2_Ce cement was approximately three times higher than that of the commercial MTA. Therefore, S_2_Ce could be considered a novel component for the hydraulic endodontic cement that provides both fast self-setting and radiopacity. The release rate of Sr ion from S_2_Ce cement was high; therefore, a bioactive effect might be expected for the released Sr ion. However, Sr dissolution is related to the hydrolysis of the cement and subsequent decrease in the compressive strength; thus, long-term in vivo stability is a concern. The relative flowability of S_2_Ce cement was lower than that of the commercial MTA, and this could be attributed to the large and irregularly shaped S_2_Ce particles. Therefore, improvement of particle shape and size control is required. Considering these properties, S_2_Ce could not be expected to be a main component of MTA. However, it can be used as an additive that exhibits fast self-setting and highly radiopaque properties.

Future studies should focus on improving the long-term stability and flowability of S_2_Ce cement by modifying the particle size and shape of S_2_Ce powders. Adhesion properties of S_2_Ce cement should also be studied as the adhesion between MTA and other restoration materials is weak [33]. Additionally, the biological effect of the Sr ion released from S_2_Ce cement should be investigated via in vitro and in vivo studies.

## 5. Conclusions

In this study, S_2_Ce was synthesized from strontium hydroxide and cerium carbonate by calcination at 1200 °C. S_2_Ce exhibited a faster setting time than commercial MTA (ProRoot MTA). In addition, it showed a higher compressive strength at 1 d after mixing than that of the commercial MTA. However, the compressive strength decreased for S_2_Ce after 28 d post mixing. Therefore, a fast setting and fast increase in the compressive strength could be expected for S_2_Ce, while its degradation is a property that requires further refinement.

S_2_Ce exhibited a large dissolution of Sr ions in water, but dissolution was not detected for Ce. This large dissolution is related to the decrease in the compressive strength of S_2_Ce. The radiopacity of S_2_Ce was three times higher than that of commercial MTA. Therefore, S_2_Ce is a possible cement component that provides a fast-setting time and sufficient radiopacity. Further studies are required to improve the long-term stability, particle shape, and size control as well as to investigate the potential biological effects. 

## Figures and Tables

**Table 1:** Dissolved ion concentrations from S_2_Ce cement (W/P = 0.4) after 1 d and 3 d immersion.

Species	1 d	3 d
Sr	5.27 ± 0.15 mg/mL (195 ± 5.5 mM)	5.68 ± 0.23 mg/mL (211 ± 8.8 mM)
Ce	not detected	not detected

## Data Availability

The data presented in this study are available on request from the corresponding author.
